# Identification of cell death-related biomarkers and immune infiltration in ischemic stroke between male and female patients

**DOI:** 10.3389/fimmu.2023.1164742

**Published:** 2023-06-26

**Authors:** Wenli Chen, Yuanfang Chen, Liting Wu, Yue Gao, Hangju Zhu, Ye Li, Xinyu Ji, Ziyi Wang, Wen Wang, Lei Han, Baoli Zhu, Hongxing Wang, Ming Xu

**Affiliations:** ^1^Department of Rehabilitation Medicine, ZhongDa Hospital Southeast University, Nanjing, China; ^2^Engineering Research Center of Health Emergency, Jiangsu Provincial Center for Disease Control and Prevention, Nanjing, China; ^3^Jiangsu Province Engineering Research Center of Health Emergency, Nanjing, China; ^4^School of Public Health, Nanjing Medical University, Nanjing, China; ^5^Department of Occupational Disease Prevention, Jiangsu Provincial Center for Disease Control and Prevention, Nanjing, China; ^6^Jiangsu Cancer Center, Jiangsu Cancer Hospital, Nanjing, China; ^7^Center for Global Health, School of Public Health, Nanjing Medical University, Nanjing, China

**Keywords:** ischemic stroke (IS), cell death, biomarker, immune infiltration, gender difference

## Abstract

**Background:**

Stroke is the second leading cause of death and the third leading cause of disability worldwide, with ischemic stroke (IS) being the most prevalent. A substantial number of irreversible brain cell death occur in the short term, leading to impairment or death in IS. Limiting the loss of brain cells is the primary therapy target and a significant clinical issue for IS therapy. Our study aims to establish the gender specificity pattern from immune cell infiltration and four kinds of cell-death perspectives to improve IS diagnosis and therapy.

**Methods:**

Combining and standardizing two IS datasets (GSE16561 and GSE22255) from the GEO database, we used the CIBERSORT algorithm to investigate and compare the immune cell infiltration in different groups and genders. Then, ferroptosis-related differently expressed genes (FRDEGs), pyroptosis-related DEGs (PRDEGs), anoikis-related DEGs (ARDEGs), and cuproptosis-related DEGs (CRDEGs) between the IS patient group and the healthy control group were identified in men and women, respectively. Machine learning (ML) was finally used to generate the disease prediction model for cell death-related DEGs (CDRDEGs) and to screen biomarkers related to cell death involved in IS.

**Results:**

Significant changes were observed in 4 types of immune cells in male IS patients and 10 types in female IS patients compared with healthy controls. In total, 10 FRDEGs, 11 PRDEGs, 3 ARDEGs, and 1 CRDEG were present in male IS patients, while 6 FRDEGs, 16 PRDEGs, 4 ARDEGs, and 1 CRDEG existed in female IS patients. ML techniques indicated that the best diagnostic model for both male and female patients was the support vector machine (SVM) for CDRDEG genes. SVM’s feature importance analysis demonstrated that SLC2A3, MMP9, C5AR1, ACSL1, and NLRP3 were the top five feature-important CDRDEGs in male IS patients. Meanwhile, the PDK4, SCL40A1, FAR1, CD163, and CD96 displayed their overwhelming influence on female IS patients.

**Conclusion:**

These findings contribute to a better knowledge of immune cell infiltration and their corresponding molecular mechanisms of cell death and offer distinct clinically relevant biological targets for IS patients of different genders.

## Introduction

As the second leading cause of mortality and the third leading cause of disability globally (1), stroke can be categorized as ischemic or hemorrhagic according to the underlying neuropathology (2). Ischemic strokes (IS) accounted for roughly 87% of all stroke occurrences. It is characterized by a sudden interruption of blood flow due to thrombosis or embolism. It obstructs the cerebral vessels supplying specific brain regions (3) and promotes a cascade of pathophysiological responses leading to immune cell infiltration and various forms of cell death (4). Stroke is distinguished by severe morbidity, death, and disability (5), with an incidence of over 13 million cases each year (6). Despite advancements in medicine, 5.5 million people died annually, and more than 50% of survivors were chronically crippled (6, 7), placing a substantial economic and illness burden on society and families (8). There is much evidence of significant differences in stroke incidence, severity, and recovery by gender, with males being more likely to have a stroke than females (9). In addition, numerous animal models of IS have demonstrated that gender differences also exist in brain injury and recovery (10). Understanding the complicated mechanisms underlying these distinctions and the various forms of cell death could provide new possibilities for discovering novel treatment targets for IS.

Existing causes of cell death mainly include ferroptosis, anoikis, pyroptosis, and cuproptosis. Ferroptosis is a form of non-apoptotic cell death that is characterized by iron overload, glutathione (GSH) depletion, glutathione peroxidase 4 (GPX4) inactivation, and an imbalance in lipid and amino acid metabolism (11), which is essential in numerous diseases, including IS and cardiovascular disease (12, 13). Some investigations have shown that MAP1LC3B, PTGS2, and TLR4 may be potential ferroptosis-related IS biomarkers (14). However, the function of ferroptosis-related genes (FRGs) in diagnosing, prognosing, or treating IS has not been fully clarified. As a unique form of apoptosis produced by separating cells from the extracellular matrix (ECM), anoikis plays a crucial role in protecting the organism, which prevents the re-adhesion of separated compartments to alternative substrates for aberrant proliferation (15). Even though some studies have proven that anoikis-related genes (ARGs) play a vital role in the tumor metastatic cascade response and cancer development (15), their essential biomarkers associated with IS have not been documented. Pyroptosis is a planned cell death related to inflammation (16), marked by cell swelling, lysis, and release of several pro-inflammatory chemicals, such as IL-1, IL-18, ATP, and HMGB1. Research shows that pyroptosis is involved in the pathogenesis of IS, and inhibition of pyroptosis can reduce ischemic brain injury (17). Interleukins (ILs) play a bidirectional function in IS via immune cell signaling, activation, and control (18). Copper can trigger several forms of cell death via multiple pathways, including apoptosis and autophagy, reactive oxygen accumulation, proteasome inhibition, and anti-angiogenesis (19). Copper toxicity causes the aggregation of mitochondrial proteins and a distinct form of cell death (20). The relationship between cuproptosis and IS has not yet been clearly explained.

Multiple immune cells penetrate across the breached blood–brain barrier from the peripheral circulation into the ischemic parenchyma, eliciting innate and adaptive immune responses (21). To date, few studies have utilized CIBERSORT to assess immune infiltration in IS. Consequently, measuring immune infiltration during the IS process is crucial for developing sophisticated targeted therapeutics. In the present study, using the estimation of relative subpopulations of RNA transcript (22), 22 infiltrating immune cells were estimated in the IS patient group and healthy control group. We downloaded a collection of four cell death-related genes from public databases and PubMed. We selected the FRDEGs, PRDEGs, ARDEGs, and CRDEGs from datasets enrolled in this study between the IS patient group and healthy control group among men and women, respectively. Finally, our team explored the relevance of these genes to immune infiltration and screened the biomarkers associated with cell death-related through four types of machine learning. This provides further ideas for future clinical research to prevent and treat IS in males and females.

## Materials and methods

### Datasets and quality control

We used “stroke” as a keyword and applied restrictions for “series” and “*Homo sapiens*” to search Gene Expression Omnibus (GEO) database files. Inclusion criteria were as follows (1): the database contains the IS patient group and healthy control group without stroke who were at least 18 years old; (2) the number of each group must be at least three; (3) with mRNA expression data of whole blood; and (4) inclusion of gender information in the clinical database. Ultimately, two data series satisfied all criteria (GSE16561 and GSE22255) (**Table 1**).

**Table 1:** Selected datasets about ischemic stroke from GEO.

GSE ID	platform	country	pubic year	healthy control	stroke patients	male	female	total
GSE16561	GPL6883	USA	2010	24	39	27	36	63
GSE22255	GPL570	Portugal	2011	20	20	20	20	40

All data and graphs in this paper were analyzed and plotted by R 4.2.1 software and Strawberry Perl 5.30.1. The data preprocessing included imputing missing expression data through KNN, removing probes without corresponding genes, taking the average when multiple probes correspond to one gene, data standardization, and then merging the two series. In total, 16,609 genes corresponding to 103 samples were ultimately enrolled. The “sva” and “limma” packages in R were used to standardize the data and eliminate the batch effect. Forty-seven male samples, namely, 27 IS patients and 20 healthy controls, had a mean age of 65.17 ( ± 12.43) years, whereas 56 additional female samples, namely, 24 IS patients and 32 healthy controls, had a mean age of 64.32 ( ± 14.12) years. There was no statistically significant difference in age between the genders (*t* = −0.32, *p* = 0.749).

### Evaluation of immune cell infiltration

The CIBERSORT algorithm was used to calculate the relative content of 22 immune cells in the male and female patient groups and in the healthy control group by Perl language. Samples with low accuracy were eliminated according to the immune infiltration results. The compositions of immune cells between the patient and healthy control groups were evaluated and compared in men and women separately. Violin plots demonstrated the results through the “vioplot” package in R.

### Extraction and analysis of CDRDEGs

The list of 484 ferroptosis genes was retrieved from the FerrDb database (http://www.zhounan.org/feroptosis/); 465 genes related to pyroptosis, 266 genes associated with anoikis, and 403 genes associated with cuproptosis (see **Supplementary Materials**) were chosen from PubMed based on published literature. The genes mentioned above were then isolated from male and female samples correspondingly. The criteria of “log_2_FC = 0.58” and “adjust-*p* = 0.05” were set, and the “limma” package in R was used to identify male and female FRDEGs, PRDEGs, ARDEGs, and CRDEGs, respectively. The “ggpubr” and “igraph” packages were applied to draw the gene box line plots and co-expression networks. Using the “RCircos” package in R, the locations of the differential genes on human chromosomes were mapped. The enrichment analyses were conducted based on those DEGs, and their associations with immune cells were investigated.

### Machine learning to identify CDRDEG biomarkers

New samples comprise FRDEGs, PRDEGs, ARDEGs, and CRDEGs expression data. Randomly dividing the samples into a training set (70%) and a test set (30%), four types of machine learning called random forest (RF), support vector machine (SVM), eXtreme gradient boosting (XGB), and generalized linear model (GLM) were used to screen the core genes, depict the model residuals, and choose disease biomarkers from CDRDEGs using machine learning models and receiver operating characteristic curve (ROC) curves to evaluate the most suitable model.

**Figure 1:**
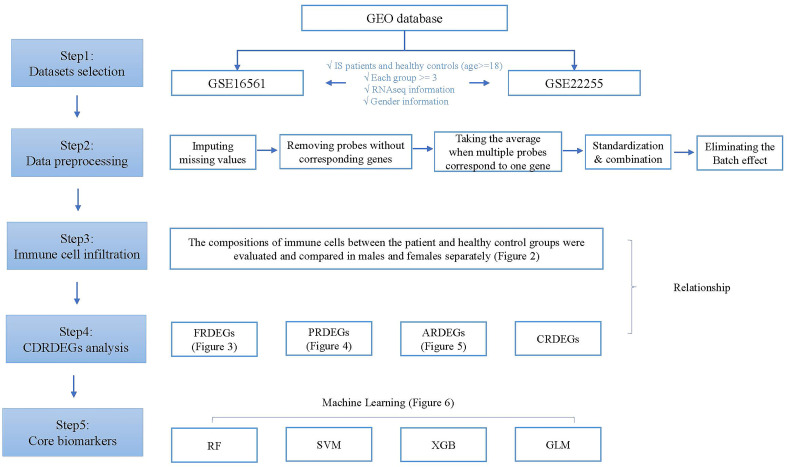
Technology roadmap.

The technology roadmap is shown in **Figure 1**.

## Results

### Immune cell infiltration in IS patients and healthy controls between men and women

**Figure 2:**
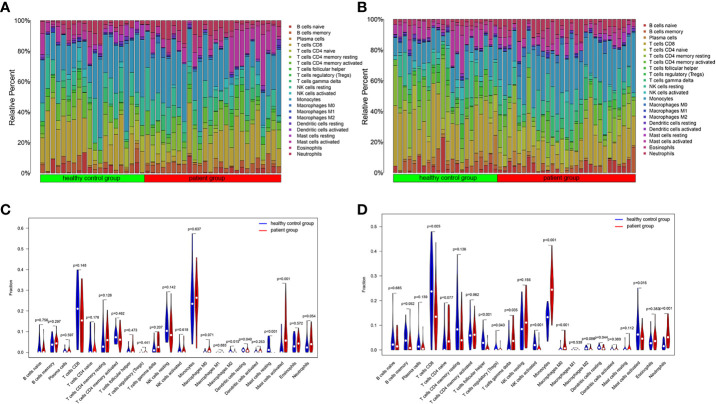
Immune cell infiltration in IS patient group and healthy control group. The relative content of 22 kinds of immune cells between IS patient group and healthy control group were showed in the histograms (Male: **A**, Female: **B**). The violin diagrams illustrated the difference in immune cell infiltration between IS patient group and healthy control group (Male: **C**, Female: **D**).

One sample was omitted from the male group because of dissatisfactory predictions (*p* > 0.05), and the infiltration results of the 22 immune cells in the other 46 samples are shown in **Figure 2A**. The violin plot (**Figure 2C**) demonstrated that the differences in the content of the four kinds of immune cells between the IS patient group and the healthy control group were statistically significant in men (*p* < 0.05). The relative amount of “Mast cells resting”, “Macrophages M2”, and “Dendritic cells resting” in the healthy control group was significantly higher than that in the patient group. In contrast, the patient group had more “Mast cells activated” than the healthy control group (**Figure 2C**; **Table 2**). Five samples were eliminated in the female group from 56 samples because of dissatisfactory predictions (*p* > 0.05) (**Figure 2B**). “T cells CD8”, “T cells follicular helper”, “T cells regulatory (Treg)”, “NK cells activated”, “Dendritic cells resting”, and “Mast cells activated” were all higher in the healthy control group compared to the patient group. In contrast, “T cells gamma delta”, “Monocytes”, “Macrophages M0”, and “Neutrophils” were lower in the healthy control group (**Figure 2D**; **Table 3**). This suggested that the altered immune cell infiltration was more active in the female patient group than in the male patient group compared to the healthy control group. “Dendritic cells resting” was the only one with lower expression in both male and female patient groups.

**Table 2:** Differential immune cells between IS patient and healthy control groups in males.

immune cell	group	mean	standard deviation	*p* value
Macrophages M2	healthy control	0.005	0.008	0.015
	patient	0.002	0.005	
Dendritic cells resting	healthy control	0.011	0.006	0.04
	patient	0.008	0.009	
Mast cells resting	healthy control	0.027	0.031	<0.001
	patient	<0.001	<0.001	
Mast cells activated	healthy control	0.027	0.043	0.001
	patient	0.095	0.092	

**Table 3:** Differential immune cells between IS patient and healthy control groups in females.

immune cell	group	mean	standard deviation	*p* value
T cells CD8	healthy control	0.238	0.129	0.005
	patient	0.142	0.097	
T cells follicular helper	healthy control	0.034	0.033	<0.001
	patient	0.008	0.015	
T cells regulatory (Tregs)	healthy control	0.008	0.02	0.040
	patient	0.001	0.007	
T cells gamma delta	healthy control	0.023	0.037	0.035
	patient	0.043	0.047	
NK cells activated	healthy control	0.023	0.023	0.001
	patient	0.007	0.017	
Monocytes	healthy control	0.121	0.045	<0.001
	patient	0.249	0.078	
Macrophages M0	healthy control	0.002	0.008	<0.001
	patient	0.022	0.027	
Dendritic cells resting	healthy control	0.011	0.007	0.044
	patient	0.007	0.007	
Mast cells activated	healthy control	0.082	0.067	0.015
	patient	0.040	0.035	
Neutrophils	healthy control	0.020	0.017	<0.001
	patient	0.061	0.042	

### Identification of ferroptosis-related biomarkers

**Figure 3:**
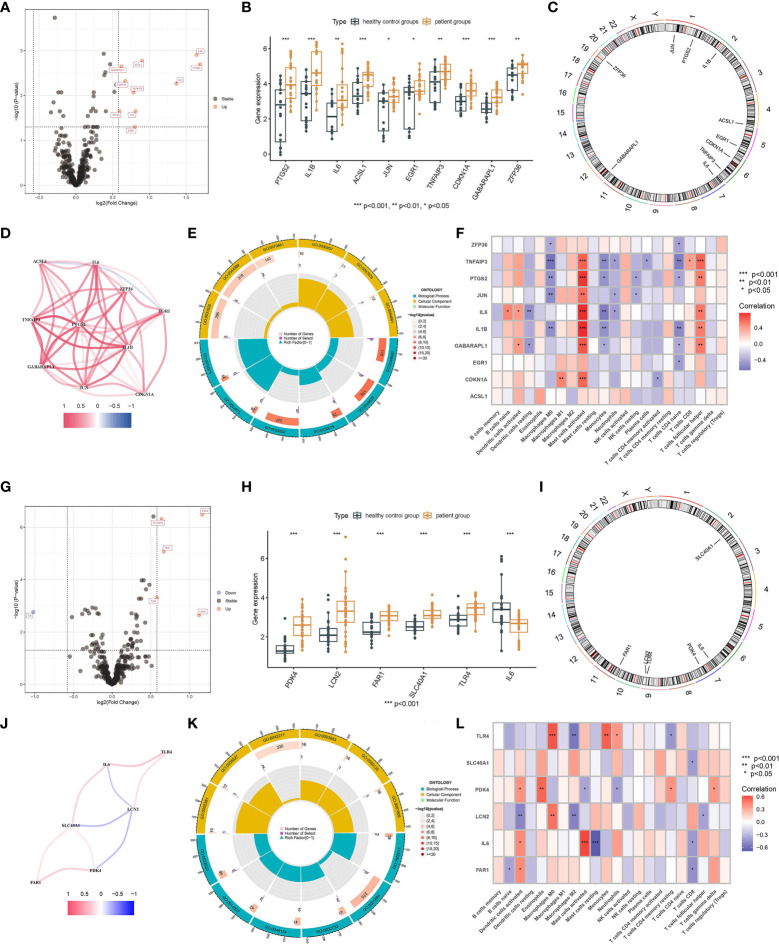
Screening of FRDEGs in the male and female groups. Volcano Plot of FRDEGs between IS patient group and healthy control group (Male: **A**, Female: **G**). BarPlot of FRDEGs between different groups (Male: **B**, Female: **H**). The distribution of FRDEGs on chromosomes (Male: **C**, Female: **I**). Co-expression network among FRDEGs(Male: **D**, Female: **J**). The main results of GO enrichment analysis of FRDEGs (Male: **E**, Female: **K**). Relationship between FRDEGs and immune cells(Male: **F**, Female: **L**).

After selecting all the FRGs, there were 10 male FRDEGs (namely IL1B, PTGS2, IL6, ACSL1, JUN, EGR1, TNFAIP3, CDKN1A, GABARAPL1, and ZFP36) between the patient group and the healthy control group, all of which were upregulated genes in IS patients (**Figures 3A, B**). Their chromosomal distribution and association are shown in **Figures 3C, D**, respectively. Those FRDEGs were mainly enriched in the pathways called “regulation of smooth muscle cell proliferation” (GO:0048660), “smooth muscle cell proliferation” (GO:0048659), and “muscle cell proliferation” (GO:0033002) (**Figure 3E**). IL6 and GABARAPL1 were negatively correlated with “Dendritic cells resting”, whereas IL6, IL1B, PTGS2, TNFAIP3, GABARAPL1, JUN, and CDKN1A were positively correlated with “Mast cells activated” in male patients (*p* < 0.05) (**Figure 3F**). There were six female FRDEGs, including PDK4, LCN2, FAR1, SLC40A1, TLR4, and IL6. All but IL6 were elevated genes in IS patients (**Figures 3G, H**). **Figure 3I** illustrates their chromosome distribution, while **Figure 3J** demonstrated their association. Those FRDEGs were chiefly enriched in the pathway of “tissue homeostasis” (GO:0001894) (**Figure 3K**). TLR4 was positively associated with “Monocytes” and “Macrophages M0” (*p* < 0.05). IL6 was positively and negatively correlated with “Mast cells activated” and “Mast cells resting”, respectively, in female patients (*p* < 0.05) (**Figure 3L**).

### Identification of pyroptosis-related biomarkers

**Figure 4:**
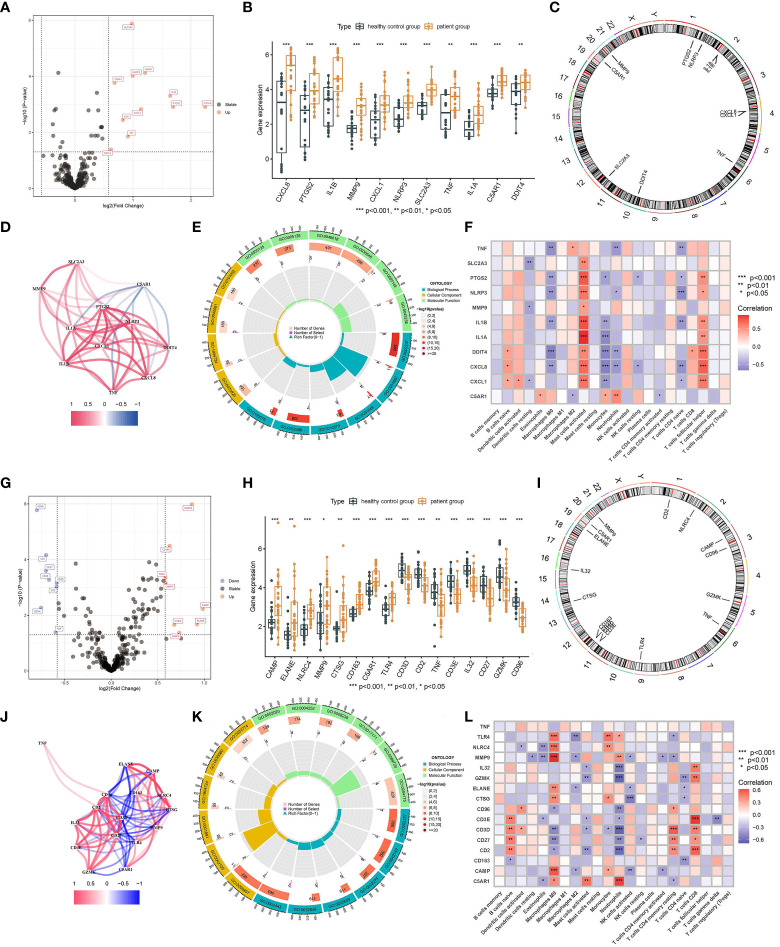
Identification of PRDEGs in different genders. Volcano Plot of PRDEGs between IS patient group and healthy control group (Male: **A**, Female: **G**). BarPlot of PRDEGs between different groups (Male: **B**, Female: **H**). The distribution of PRDEGs on chromosomes (Male: **C**, Female: **I**). Co-expression network among PRDEGs (Male: **D**, Female: **J**). The main results of GO enrichment analysis of PRDEGs (Male: **E**, Female: **K**). Relationship between PRDEGs and immune cells(Male: **F**, Female: **L**).

There were 11 upregulated male PRDEGs in the patient group compared with the healthy control group, namely CXCL8, PTGS2, IL1B, MMP9, CXCL1, NLRP3, SLC2A3, TNF, IL1A, C5AR1, and DDIT4 (**Figures 4A, B**). Their chromosomal distribution and co-expression network are shown in **Figures 4C, D**. Those PRDEGs were primarily enriched in the pathways called “response to molecule of bacterial origin” (GO:0002237) and “response to lipopolysaccharide” (GO:0032496) (**Figure 4E**). PTGS2, NLRP3, IL1A, IL1B, DDIT4, CXCL8, and CXCL1 were positively connected with “Mast cells activated” and “T cells follicular helper”, but negatively correlated with “Monocytes” except for NLRP3 (*p* < 0.05) (**Figure 4F**). There were 16 female PRDEGs, of which CAMP, ELANE, NLRC4, MMP9, CTSG, CD163, TLR4, and C5AR1 were upregulated genes, and CD96, GZMK, IL32, CD27, CD3E, CD3D, CD2, and TNF were downregulated genes in IS patients (**Figures 4G, H**). Their distribution on chromosomes was depicted in **Figure 4I**, and the co-expression network is illustrated in **Figure 4J**. Those PRDEGs were mainly enriched in the pathways called “response to molecule of bacterial origin” (GO:0002237), “defense response to bacterium” (GO:0042742), and “leukocyte mediated immunity” (GO:0002443) (**Figure 4K**). Those PRDEGs were mainly related to “Macrophages M0”, “Neutrophils”, “T cells CD4 memory resting”, and “T cells CD8” (*p* < 0.05) (**Figure 4L**).

### Identification of anoikis-related biomarkers

**Figure 5:**
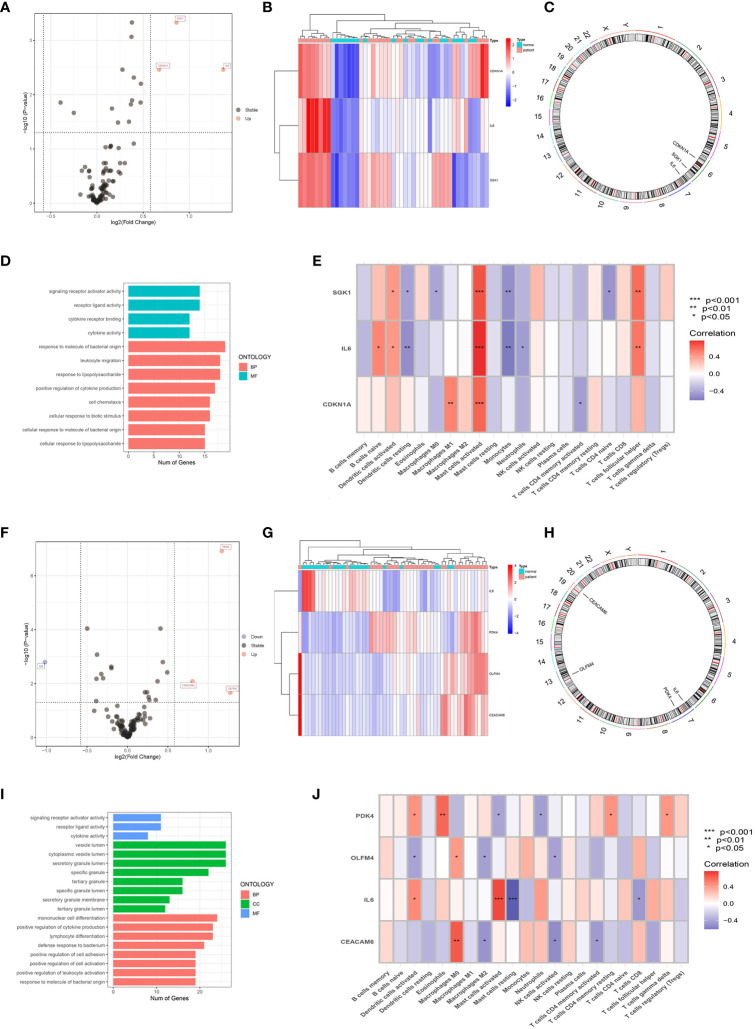
Screening of ARDEGs in the male and female groups. Volcano Plot of ARDEGs between IS patient group and healthy control group (Male: **A**, Female: **F**). Heatmap of ARDEGs between different groups (Male: **B**, Female: **G**). The distribution of ARDEGs on chromosomes (Male: **C**, Female: **H**). The main results of GO enrichment analysis of ARDEGs (Male: **D**, Female: **I)**. Relationship between ARDEGs and immune cells (Male: **E**, Female: **J**).

Three male ARDEGs (IL6, SGK1, and CDKN1A) were upregulated in IS patients (**Figures 5A, B**) and located on chromosomes 6 and 7 (**Figure 5C**), chiefly concentrating on the pathway of “response to molecule of bacterial origin” (**Figure 5D**). **Figure 5E** demonstrated that IL6 and SGK1 were negatively connected with “Dendritic cells resting” and “Monocytes”, but IL6, SGK1, and CDKN1A were positively correlated with “Mast cells activated” (*p* < 0.05). There were four female ADEGs (namely OLFM4, PDK4, CEACAM6, and IL6), all of which were elevated genes except for IL6 (**Figures 5F, G**). Their location on chromosomes is shown in **Figure 5H**, and their most important GO channels are depicted in **Figure 5I**. IL6 was positively and negatively correlated with “Mast cells activated” and “Mast cells resting”, while OLFM4 and CEACAM6 were strongly positively correlated with "Macrophages M0" and negatively correlated with "Macrophages M2" and "NK cells activated" (**Figure 5J**).

### Identification of cuproptosis-related biomarkers

There were only one upregulated and one downregulated cuproptosis-related DEGs (CRDEGs) in male and female patients, called NLRP3 and MT1X, respectively.

### Machine learning of various CDRDEG biomarkers

**Figure 6:**
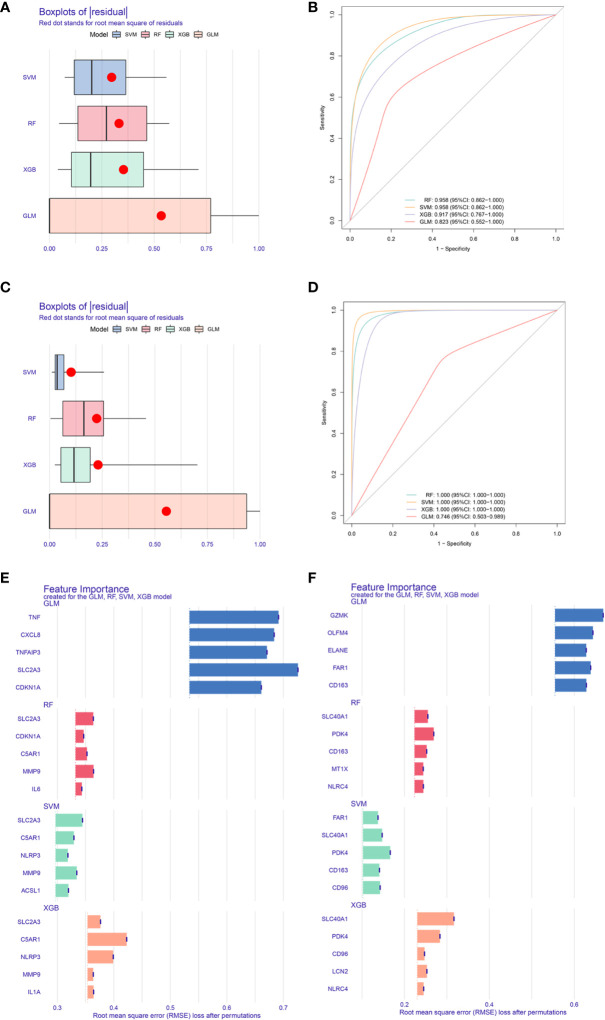
Core biomarkers related to cell death and selected by machine learning. Boxplots of residual for four models(Male: **A**, Female: **C**). ROC for four models (Male: **B**, Female:**D**). Feature importance created by four models (Male: **E**, Female: **F**).

Above all, we got a set of 20 male and 24 female CDRDEGs, including FRDEGs, PRDEGs, ARDEGs, and CRDEGs (**Table 4**). RF, SVM, XGB, and GLM were used to screen biomarkers (**Figures 6A–D**). The findings demonstrated that SVM had the best ROC efficiencies and the minor residues in both male and female groups. Following the SVM outcomes, SLC2A3, MMP9, C5AR1, ACSL1, and NLRP3 were the top five feature-important CDRDEGs in male IS patients. Meanwhile, the PDK4, SCL40A1, FAR1, CD163, and CD96 displayed their overwhelming influence in female IS patients (**Figures 6E, F**).

**Table 4:** Cell death-related DEGS between IS patient and healthy control groups with different genders.

Gene	Male				Female			
	FRDEG	PRDEG	ARDEG	CRDEG	FRDEG	PRDEG	ARDEG	CRDEG
IL1B	↑	↑						
PTGS2	↑	↑						
IL6	↑		↑		↓		↓	
ACSL1	↑							
JUN	↑							
EGR1	↑							
TNFAIP3	↑							
CDKN1A	↑		↑					
GABARAPL1	↑							
ZFP36	↑							
PDK4					↑		↑	
LCN2					↑			
FAR1					↑			
SLC40A1					↑			
TLR4					↑	↑		
SGK1			↑					
OLFM4							↑	
CEACAM6							↑	
CXCL8		↑						
MMP9		↑				↑		
CXCL1		↑						
NLRP3		↑		↑				
SLC2A3		↑						
TNF		↑				↓		
IL1A		↑						
C5AR1		↑				↑		
DDIT4		↑						
CAMP						↑		
NLRC4						↑		
ELANE						↑		
CTSG						↑		
CD163						↑		
CD96						↓		
GZMK						↓		
IL32						↓		
CD27						↓		
CD3E						↓		
CD3D						↓		
CD2						↓		
MT1X								↓

↑: Up-graduated gene; ↓: Down-graduated gene.

## Discussion

Despite significant advances in the early diagnosis and treatment of IS over the past decade, stroke remains a leading cause of mortality and long-term disability, placing a significant burden on human health and the economy. There are significant differences in morbidity, mortality, and outcome of IS between males and females, understanding the causes of which is crucial for effective diagnosis and treatment (9). It suggests that we can investigate potential therapeutic targets of IS from a gender perspective separately.

Multiple immune cells can infiltrate the brain parenchyma orderly after an acute stroke. The peripheral immune cells, including myeloid dendritic cells, monocytes/macrophages, and neutrophils, appear within 1 day after stroke, peak at 3 days, and continue until 7 days, followed by a modest rise in T and B lymphocytes (23). The results of immune cell infiltration in male and female patients showed that immune cell infiltration was more active in female IS patients than male IS patients. Only Dendritic cells resting, an immune cell, were expressed at low levels in male and female patients. Dendritic cells are known to be the most efficient antigen-presenting cells (APCs). They act as outposts of the immune system (including the central nervous system), constantly monitoring the environment and playing a crucial linkage role between innate and adaptive immunity (24). The presence of DCs in the brain parenchyma is usually rare. However, as the neuroinflammatory response increases, the resting state of DCs is activated, leading to an increase in DCs in the brain parenchyma and a decrease in Dendritic cells resting (23). Consistent with our findings, Dendritic cells resting are expressed at modest levels in both male and female patients. In our investigation, the difference in the mast cell content between resting and activated states was significant in the male patient group and the healthy control group. Numerous investigations have linked mast cells to multiple neuroinflammatory disorders of the central nervous system, including stroke (25). Within a few hours following IS, mast cells are transformed from a resting state to an active state and then generate an adaptive immune response by activating T and B lymphocytes, and a subgroup of T cells can aid or aggravate ischemic brain damage (26). In the female population, the difference in monocyte content is more significant. Within 24 h following an acute ischemic stroke (AIS), peripheral monocytes infiltrate the lesion site, with a peak between 3 and 7 days, which have been believed to affect AIS negatively (27). Monocytes are connected with inflammation because they synthesize inflammatory chemicals upon activation (such as upon stimulation of Toll-like receptors) (28). Thus, the patient group has much greater levels of monocytes than the healthy control group in women.

There is mounting evidence that inflammatory cells and the immune system play a crucial role in the development of stroke. Targeted interventions based on immunoinflammatory IS therapies, and cell death-related biomarkers may offer novel diagnostic and therapeutic options for IS (29–32). In combination with residual plots and ROC curves, we screened the relevant differential genes of the four modes of cell death, including ferroptosis, anoikis, pyroptosis, and cuproptosis. Then, we selected the best-fitting model to obtain the five most significant biomarkers selected by this model. The five most relevant male biomarkers identified were SLC2A3, MMP9, C5AR1, ACSL1, and NLRP3. Except for ACSL1, a biomarker associated with ferroptosis, SLC2A3, MMP9, C5AR1, and NLRP3 are all biomarkers associated with pyroptosis. It has been shown that SLC2A3 is co-expressed with MMP9, SLC2A3 is co-expressed with C5AR1, and C5AR1 is co-expressed with NLRP3 (33–35), and they are all elevated genes in the male IS patient group. Therefore, pyroptosis is the primary form of cell death determining the course of IS in male patients. PDK4, SCL40A1, FAR1, CD163, and CD96 were chosen as the five most essential relevant biomarkers in female patients based on the best model. CD163 and CD96 are biomarkers connected to pyroptosis, SCL40A1 and FAR1 are biomarkers associated with ferroptosis, and PDK4 is a biomarker related to anoikis. In contrast to male patients, ferroptosis and pyroptosis are primarily responsible for advancing IS in female patients. It is undeniable that cellular scorch death, a programmed cell death associated with inflammation, plays a crucial role in the development of IS in both male and female patients (36). The assembly and activation of NLRP3 inflammatory vesicles might lead to cystathione-1-dependent release of pro-inflammatory cytokines such as IL-1β and IL-18. Therefore, NLRP3 expression is elevated in IS patients (37). Existing research indicates that several inhibitors of localized death targeting cystein, NLRP3, and upstream pathways could minimize stroke-related brain tissue damage (33). In addition, we determined that the positive connection between active mast cells and NLRP3 was very significant. During innate immunity in response to IS, NLRP3 inflammatory vesicles produce a variety of pro-inflammatory cytokines that cleave pro-interleukin-1 (pro-IL-1) into its active form in mast cells, thereby mediating neuronal cell dysfunction and brain edema and, ultimately, neuronal cell death (38, 39). This is further evidence of the importance of NLRP3; hence, we can examine innovative therapy strategies for IS from an NLRP3 perspective. We also revealed in this work that SLC2A3 expression was increased in IS. The involvement of SLC2A3 in the SLC2A3–STAT3–SLC2A3 feedback loop can activate the STAT3 signaling pathway and phosphorylation of downstream glycolytic target genes. The most important function of SLC2A3 is to increase immunological infiltration and bias TAM polarization toward an M2-like phenotype (40). Ablation of MMP9 inhibits high glucose-induced apoptosis and localized death in human cardiac stem cells (HCSCs). The ROS-induced SAPK/JNK pathway, which promotes oxidative stress and cell death, may be the primary signaling mechanism for MMP9-mediated HCSC death. The lack of MMP9 inhibits apoptosis and anoikis in normal glucose HCSCs (41). Our results also indicate that MMP9 was significantly expressed in IS and that reduction of MMP9 activity may be useful for preventing apoptosis and functioning as a therapeutic agent for the disease. C5A receptor 1 (C5AR1) induces a strong inflammatory response to injury. Rat neuronal cells in a model of cerebral ischemia and reperfusion (I/R) exhibit significant upregulation of C5aR1 gene expression, and inflammatory cascade signals, including TLR4, TNF-α, IL-1β, and IL-6, are regulated in parallel with the regulation of C5AR1 expression levels, demonstrating the pathogenic role of C5AR1 in the progression of brain injury and inflammatory response after I/R injury (42). The results of this study corroborated the function of C5AR1 as a gene that was elevated in IS. As a possible biomarker and therapeutic target for inflammation, CD163 expression is strongly induced by anti-inflammatory mediators such as glucocorticoids and IL-10, while it is simultaneously inhibited by pro-inflammatory mediators such as interferon-γ (43). The soluble plasma CD163 could be considered as a biomarker caused by increased CD163 shedding mediated by tumor necrosis factor-α (TNF-α) lyase. Consistent with the elevation of CD163 expression in the result, elevated CD163 expression in IS indicates tissue response to inflammation. CD96 is critical for immune function regulation, which participates in different immunological responses, controls immune cell infiltration, and affects the malignant properties of various cancer types (44). Consequently, in contrast to other indicators, CD96 was downregulated in IS patients, significantly impacting immune cell infiltration. In addition, our result that mast cells in an active state were negatively connected with CD96 concerning immune cells and differential gene expression further validates the significance of CD96 in IS. Increasing CD96 expression may effectively suppress mast cell activation, reducing disease development and presenting a unique therapeutic target for illness therapy. In ferroptosis, it has been found that SFA-mediated ferroptosis is dependent on FAR1 by converting SFA to fatty alcohols, while SCL40A1, an iron transporter protein, also plays an important role and was upregulated in IS patients (45). Inactivation of FAR1 and SCL40A1 promotes cell resistance to ferroptosis, providing a new possibility for treating IS.

With the present findings, we found that gene expression was significantly different between male and female IS patients and that gender factors influenced the primary mode of cell death. Previous studies suggest that ischemia-induced cell death pathways may differ in male and female patients, with male cell death triggered by poly(ADP-ribose) polymerase (PARP) activation and nuclear translocation of apoptosis-inducing factor (AIF), and that interference with this pathway is beneficial for male patients with IS but not for female patients. In contrast, cystathionin activation may be the main pathway activated after ischemic injury in female patients (46). The genetic female and male cell responses to cerebral ischemia are also mechanistically different, which also suggests that molecular signaling pathways activated by ischemia may differ in the male and female brains (47). These differences may be related to specific physiological factors in male and female patients. The female-specific natural estrogens have anti-atherogenic and neuroprotective effects and play a protective role during the female reproductive years, with a low-risk profile for atherosclerosis before menopause and an increased incidence of these diseases after menopause due to the loss of estrogenic protection (48). Results of studies on the relative contribution of chromosomes X and Y to stroke outcome suggest that ischemic susceptibility is mediated exclusively by the action of circulating gonadotropic hormones (49). In global models of cerebral ischemia after cardiac arrest, the presence of circulating estradiol in female mice has a relative protective effect against ischemic injury, and molecular signaling may involve estrogen receptor subtype B or G protein-coupled receptor 30, resulting in less histological injury in female mice compared to male mice (50). As is well-known, minocycline only improved stroke outcomes and serum uric acid levels in male patients. The interaction on infarct growth was significant only in female patients (51). Other clinical findings demonstrate the importance of considering gender as a biological variable when exploring potential therapeutic targets for IS. Recognizing the numerous physiological variations between males and females guarantees that the design and dynamics of our studies can be analyzed for gender-specific outcomes and targeted to screen for potential therapeutic targets across gender.

There are some limitations in this study. Firstly, more research must be conducted on the molecular processes of the selected significant biomarkers in IS. Secondly, our study’s data were gathered from internet databases; further studies with large sample sizes or in-depth functional investigations will be helpful for a better understanding of our findings. In future research, we must continue refining this concept to give a more accurate and conclusive framework for investigating new therapies for IS patients in both men and women.

## Conclusion

In conclusion, a thorough bioinformatics study of DEGs and pathways implicated in the onset and progression of IS-relative immune cell infiltration was conducted. Critical regulatory genes and pathways leading to cell death were investigated and identified. In addition, these findings may facilitate the comprehension of molecular pathways and therapeutically relevant molecular targets for the rapid identification of IS in first aid, and give fresh insight into the prevalence and progression of IS in different genders.

## Data availability statement

The datasets presented in this study can be found in online repositories. The names of the repository/repositories and accession number(s) can be found in the article/**Supplementary Material**.

## Author contributions

WC, YC, and LW performed most of data analysis and contributed in writing the manuscript. HZ, XJ, YL, and WW performed database search job. YG and ZW contributed in data pre-processing. HW and MX conceived the overall scope of the project and wrote the manuscript. WC, HW, BZ, and LH provide the funding support. All authors contributed to the article and approved the submitted version.
